# *Peribacillus suis* sp. nov. Isolated From the Pig Louse *Haematopinus suis* Reveals Unexpected Pathogenic Potential in a Traditionally Benign Genus

**DOI:** 10.1155/tbed/8640992

**Published:** 2026-03-27

**Authors:** Yan-Yan Peng, Hany M. Elsheikha, Ying Xun, Yi-Liu Liu, Ya Zhang, Yuan-Ping Deng, Shi-Feng Hu, Yi-Tian Fu, Guo-Hua Liu

**Affiliations:** ^1^ Research Center for Parasites and Vectors, College of Veterinary Medicine, Hunan Agricultural University, Changsha, 410128, Hunan Province, China, hunau.edu.cn; ^2^ Faculty of Medicine and Health Sciences, School of Veterinary Medicine and Science, Sutton Bonington Campus, University of Nottingham, Loughborough, LE12 5RD, UK, nottingham.ac.uk; ^3^ College of Animal Science and Technology, Tarim University, Alaer, 843300, Xinjiang, China, taru.edu.cn

**Keywords:** comparative genomics, emerging pathogenicity, *Haematopinus suis*, *Peribacillus suis* sp. nov., polyphasic taxonomy

## Abstract

The pig sucking louse *Haematopinus suis* is a major swine ectoparasite and vector of pathogens, yet its microbiome remains understudied. Here, we described the isolation of a previously unrecognized bacterial strain, P8‐9^T^, from the intestinal tract of *H. suis*. A comprehensive polyphasic analysis, including phenotypic characterization, phylogenomics, and whole‐genome comparisons, placed this isolate within *Peribacillus*. Genome‐based metrics confirmed its novelty, with average nucleotide identity and digital DNA–DNA hybridization values (<80% and <27%, respectively) falling far below accepted species delineation thresholds. We, therefore, proposed the designation *Peribacillus suis* sp. nov. Unexpectedly, the genome of P8‐9^T^ encodes an extensive repertoire of 219 putative virulence‐associated and antibiotic resistance genes; features atypical for a largely composed of environmental species considered saprophytic or beneficial. In vivo experiments revealed this pathogenic potential: intraperitoneal inoculation in mice resulted in all animals reaching predefined humane endpoints within 24 h, characterized by septicemia and widespread organ pathology, while oral exposure elicited splenomegaly, intestinal pathology, and a robust pro‐inflammatory response. This work represents the first report of a *Peribacillus* species isolated from an ectoparasite and provides direct experimental evidence of virulence within a genus traditionally viewed as benign.

## 1. Introduction

Blood‐feeding arthropods are well‐recognized vectors of infectious agents, yet their resident microbiomes remain an underexplored frontier for pathogen discovery [1–3]. These microbial communities, shaped by the distinct selective pressures of a hematophagous lifestyle, may harbor bacteria with unexpected ecological roles and pathogenic potential [4]. The pig sucking louse, *Haematopinus suis*, is a globally distributed ectoparasite responsible for transmitting and carrying multiple swine pathogens and imposing substantial economic losses on the pig industry [5, 6]. Our previous metagenomic analysis revealed that the gut microbiome of *H. suis* is both diverse and largely uncharacterized [7], positioning this ectoparasite as a promising reservoir for discovering novel microorganisms with unique biological functions.

Building directly on these findings, our prior metagenomic survey of the *H. suis* gut microbiome [7] not only revealed its high microbial diversity but also identified sequence reads with high homology to members of the genus *Peribacillus*. This preliminary genomic signal, detected within a blood‐feeding ectoparasite, presented a compelling ecological anomaly given the generic established association with soil and plants. We, therefore, strategically targeted this bacterial group for cultivation and in‐depth characterization.

Within this broader microbial landscape, the genus *Peribacillus* has historically been defined by a benign ecological profile. Since its separation from the *Bacillus* complex, *Peribacillus* has been dominated by environmental and plant‐associated species widely recognized for saprophytic or plant‐growth‐promoting properties [8, 9]. This perspective is reinforced by extensive reports of *Peribacillus* isolates from soil and rhizosphere habitats, where they contribute to nutrient cycling and biological control [10, 11]. However, emerging evidence challenges this paradigm. Even environmental *Peribacillus* strains have been found to encode virulence‐associated and antibiotic resistance determinants alongside their beneficial traits [12]. Furthermore, clinical reports implicating *Peribacillus simplex* in opportunistic human infections [13, 14] suggest that pathogenic traits within this genus may be more widespread than previously recognized. Collectively, these observations indicate the possibility of a broader, cryptic pathogenic potential within *Peribacillus*.

The emergence of pathogenicity among bacteria traditionally regarded as environmental is increasingly documented across diverse taxa [15, 16]. Yet, the absence of targeted investigations into *Peribacillus* species from animal‐associated or parasitic niches has left this potential largely unexplored. In this study, we address this knowledge gap by reporting the discovery of *Peribacillus suis* sp. nov., a novel species isolated from the gut of *H. suis*. Using a comprehensive polyphasic framework, including genome‐based taxonomic analyses [17, 18], we establish its distinct species status. Comparative genomic analyses revealed that *P. suis* harbors an unexpectedly extensive repertoire of putative virulence factors and antibiotic resistance genes; features atypical for the genus. More critically, we demonstrated its pathogenicity in vivo. Systemic inoculation in a mouse model induced acute, lethal septicemia, while oral challenge produced pronounced intestinal lesions and a strong pro‐inflammatory response.

These findings provide the first experimental evidence of severe pathogenicity within the genus *Peribacillus*, fundamentally challenging its long‐standing characterization as benign. This work extends the ecological scope of *Peribacillus* to include an arthropod‐associated niche and highlights the value of exploring neglected ectoparasite microbiomes as reservoirs for microorganisms with emerging relevance to animal health.

## 2. Methods

### 2.1. Ethics Approval and Consent to Participate

All mouse experiments were conducted in accordance with the ARRIVE 2.0 guidelines. The study was approved by the Animal Research Ethics Committee of Hunan Agricultural University (Approval No. 202282).

### 2.2. Isolation and Culture Conditions

Adult *H. suis* were collected from domestic pigs in Sichuan Province, China. To minimize surface contamination, live lice were subjected to a surface disinfection procedure by brief immersion in 75% ethanol, followed by three rinses in sterile phosphate‐buffered saline (PBS). All subsequent procedures were performed under aseptic conditions. The stereomicroscope and workspace were wiped with 75% ethanol and exposed to UV light in a biosafety cabinet for 15–30 min prior to use. Subsequently, the intestinal tracts were aseptically dissected from the surface‐sterilized lice under the stereomicroscope within the biosafety cabinet. The intestinal tissues were weighed and homogenized in sucrose–phosphate–glutamate (SPG) buffer. The homogenate was serially diluted to 10^−5^, subjected to heat treatment at 65°C for 30 min, and inoculated into aerobic blood culture bottles. After 5 days of pre‐incubation, 50 µL of the supernatant was spread onto Schaedler agar plates and incubated aerobically at 37°C for 24 h. A single colony, designated strain P8‐9^T^, was isolated, purified, and preserved for subsequent analyses.

### 2.3. Phenotypic Characterization

Purified colonies of the F1 generation were picked with a sterile inoculating loop and inoculated into LB liquid medium, followed by static incubation at 37°C for 18 h to prepare seed cultures. Subsequently, the seed cultures were used to perform two sets of cultivation experiments using a 1% (v/v) inoculation ratio. The first set was inoculated into LB liquid medium and incubated with shaking at 220 r/min for 24 h at various temperatures (15, 20, 25, 30, 37, and 45°C). The second set was inoculated into LB medium adjusted to different NaCl concentrations (0%–20% w/v) or pH values (4–13) and incubated at 37°C and 220 r/min for 24 h. Finally, growth under the different conditions was compared by measuring the optical density at 600 nm (OD_600_). Colony and cellular morphologies were assessed after 24 h of incubation at 37°C. Gram staining was performed and biochemical traits were assessed using standard microbiological procedure.

### 2.4. Phylogenetic and Genomic Analysis

Matrix‐assisted laser desorption/ionization time‐of‐flight mass spectrometry (MALDI‐TOF MS) was applied for initial bacterial identification, as it is considered a highly efficient alternative to 16S rRNA sequencing [19, 20]. For strain P8‐9^T^, MALDI‐TOF MS analysis yielded a log(score) of 1.93, which is below the threshold of 2.0 required for reliable species‐level identification and suggests the strain was not represented in the database [21]. Genomic DNA was subsequently extracted using the Tianamp Bacteria DNA Kit (Tiangen, China). The nearly full‐length 16S rRNA gene was amplified using universal primers 27F and 1492R and sequenced. Resulting sequences were compared with the EzBioCloud and NCBI databases.

Multiple sequence alignment was performed with MAFFT v7.471, and phylogenetic relationships were inferred using the maximum‐likelihood (ML) method in MEGA 12 under the K2 + G + I substitution model, selected as the best fit. Branch support was evaluated with 1000 bootstrap replicates.

Genomic DNA was sent to Novogene (Beijing, China) for whole‐genome sequencing and assembly. Whole‐genome sequencing employed a hybrid approach combining Illumina PE150 and Oxford Nanopore PromethION reads. De novo assembly was performed using Unicycler, yielding a complete circular chromosome. Gene prediction and annotation were conducted using GeneMarkS [22], tRNAscan‐SE [23], and rRNAmmer [24]. Functional annotations were assigned by BLASTP alignment against the nonredundant (NR) protein database (https://ftp.ncbi.nih.gov/blast/db/), Kyoto Encyclopedia of Genes and Genomes (KEGG, http://www.genome.jp/kegg/), Gene Ontology databases (GO, http://www.geneontology.org/), and pathogen–host interactions database (PHI‐base, http://www.phi-base.org/). Putative virulence factors and antibiotic resistance genes were specifically screened against the virulence factor database (VFDB, http://www.mgc.ac.cn/VFs/main.htm) and the comprehensive antibiotic resistance database (CARD, https://card.mcmaster.ca/), respectively. For these functional annotations, we applied a cutoff of ≥40% amino acid identity and ≥70% query coverage. Average nucleotide identity (ANI) was calculated using the OAT tool on the EzBioCloud platform [25], and digital DNA–DNA hybridization (dDDH) estimates were obtained using the GGDC web service [26].

### 2.5. Mouse Pathogenicity Experiments

All animal experiments were conducted in accordance with ARRIVE 2.0 guidelines for the care and use of laboratory animals. Specific‐pathogen‐free (SPF) Kunming mice (20 ± 2 g) were obtained from Hunan SJA Laboratory Animal Co., Ltd. Mice were randomly assigned to three groups: a control group (untreated), an oral gavage (GW) group, and an intraperitoneal (IP) injection group, with *n* = 10 per group for survival analysis and *n* = 3 per group per time point for histopathology and quantitative PCR (qPCR).

The inoculum administered to the GW and IP groups contained 1 × 10^8^ CFU of strain P8‐9^T^, a dose established through pilot studies and consistent with infection models used for other opportunistic pathogens [27]. Mice were monitored for 10 days for clinical signs and predefined humane endpoints. For histopathology, mice were euthanized at 3, 9, and 24 h post‐infection via CO_2_ asphyxiation following anesthesia with Zoletil 50 (30 mg/kg), in accordance with American Veterinary Medical Association (AVMA) guidelines. Organs were collected (liver, lung, spleen, and duodenum for GW; liver, lung, and spleen for IP), fixed in 4% paraformaldehyde, processed, sectioned, and stained with hematoxylin and eosin (H&E) following standard protocols.

For gene expression analysis, total RNA was extracted from snap‐frozen tissues using TRIzol reagent following established single‐step RNA isolation procedures [28]. cDNA was synthesized using Evo M‐MLV Reverse Transcriptase Reagent Premix (Hunan Accurate Biotechnology Co., Ltd., Changsha, China). qPCR was performed using PerfectStart Green qPCR SuperMix (TransGen Biotech Co., Ltd., Beijing, China) to detect the transcriptional expression levels of three target genes, namely interleukin‐1β (IL‐1β), tumor necrosis factor‐α (TNF‐α), and transforming growth factor‐β1 (TGF‐β1). Primer sequences are provided in Table 1. *gapdh* served as the internal reference gene, and relative gene expression was calculated using the 2^−ΔΔCt^ method [29].

**Table 1:** Primers used for amplification of target genes.

Name	Primer sequence (5′–3′)	Primer length (bp)
GAPDHF	AGAAGGTGGTGAAGCAGGCATC	22
GAPDHR	CGAAGGTGGAAGAGTGGGAGTTG	23
IL‐1βF	CTCGCAGCAGCACATCAACAAG	22
IL‐1βR	CCACGGGAAAGACACAGGTAGC	22
TNF‐ɑF	ACGCTCTTCTGTCTACTGAACTTCG	25
TNF‐ɑR	TGGTTTGTGAGTGTGAGGGTCTG	23
TGF‐β1F	TGGAGCAACATGTGGAACTC	20
TGF‐β1R	GTCAGCAGCCGGTTACCA	18

### 2.6. Statistical Analysis

Data analysis was performed using IBM SPSS Statistics 27 and GraphPad Prism 9.0. Differences in cytokine expression between groups were assessed using one‐way analysis of variance (ANOVA) followed by Tukey’s honest significant difference (HSD) post hoc test. A probability value (*p*‐value) <0.05 was considered statistically significant.

## 3. Results

### 3.1. Phenotypic and Chemotaxonomic Characteristics

Strain P8‐9^T^ is a Gram‐positive, facultatively anaerobic, and short‐rod–shaped bacterium (Figure 1A). After 24 h of incubation on Schaedler agar at 37°C, it formed light yellow, circular colonies with irregular margins (Figure 1B). The strain grew over a broad range of temperatures (15–45°C; optimum 37°C), pH values (5–10; optimum pH 7), and NaCl concentrations (0%–10% w/v; optimum 1%) (Supporting Information 1: Figure S1). Phenotypic comparison with its closest relatives revealed that strain P8‐9^T^ exhibited an expanded carbohydrate utilization profile, including galactose, glucose, fructose, mannose, mannitol, amygdalin, esculin, cellobiose, maltose, sucrose, and trehalose (Table 2).

Figure 1Morphology and Gram character of *Peribacillus suis* sp. nov. (A) Gram staining of *P. suis* sp. nov strain P8‐9^T^. (B) Colony morphology of *P. suis* sp. nov strain P8‐9^T^.

**Figure:**
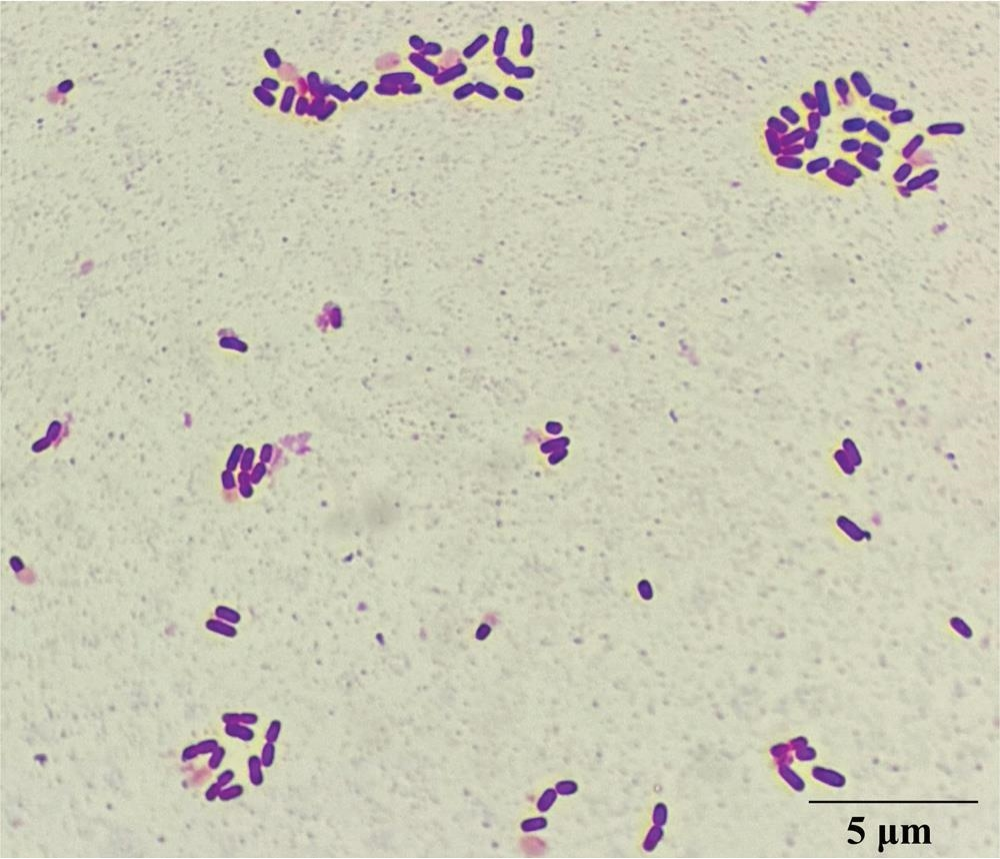
(A)

**Figure:**
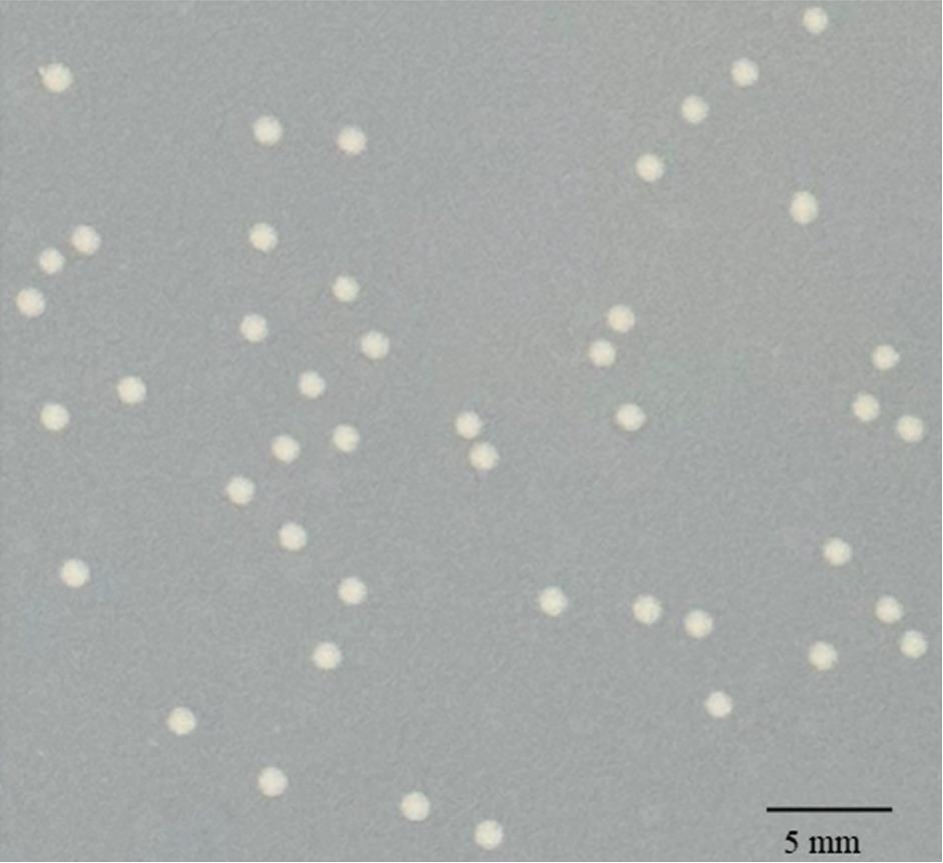
(B)

**Table 2:** Differential characteristics between strain P8‐9^T^ and its closest related species.

Characteristic	1	2	3	4	5	6	7
Amygdalin	+	−	−	+	−	+	w
Cellobiose	+	−	−	−	+	−	−
Erythritol	−	−	−	−	−	−	−
Esculin	+	−	−	nd	−	−	−
Fructose	+	−	−	−	+	+	−
Galactose	+	−	−	−	−	+	−
Glucose	+	+	−	−	+	+	−
Inulin	−	−	−	−	+	−	+
Lactose	−	−	−	−	−	+	−
Maltose	+	−	−	−	+	−	−
Mannitol	+	−	−	−	+	+	−
Mannose	+	−	−	−	+	+	−
Melibiose	−	−	−	−	−	−	−
Ribose	−	−	−	−	−	+	−
Salicin	−	−	+	nd	−	−	−
Sucrose	+	−	−	−	+	−	−
Trehalose	+	−	−	−	+	−	−

*Note:* Strain 1, strain P8‐9^T^; strain 2, *P. aracenensis* BBB004^T^; strain 3, *P. simplex* DSM 1321^T^; strain 4, *P. castrilensis* N3^T^; strain 5, *P. frigoritolerans* DSM 8001^T^; strain 6, *P. muralis* DSM 16288^T^; strain 7, *P. butanolivorans* DSM 18926^T^. Symbols: ‘‘+,” positive; ‘‘−,” negative.

Abbreviations: nd, not determined; w, weak.

### 3.2. Phylogenetic Position and Genomic Delineation of a Novel Species

**Figure 2:**
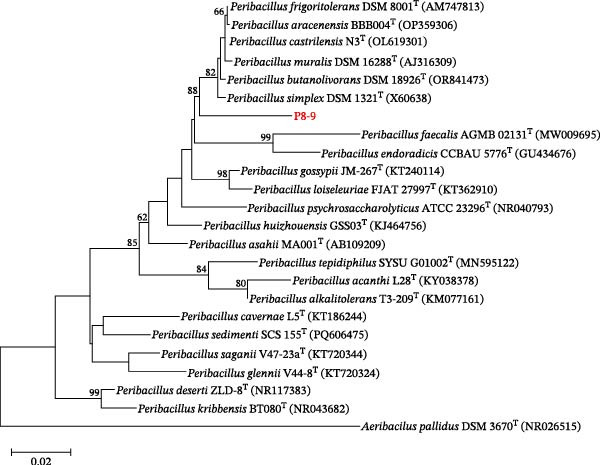
16S rRNA–based phylogenetic position of *Peribacillus suis* sp. nov. Phylogenetic analysis based on 16S rRNA gene sequences showing the position of *P. suis* sp. nov. strain P8‐9^T^ within the genus *Peribacillus*. Species in red font is *P. suis* sp. nov isolated and named in the present study.

MALDI‐TOF MS yielded a log(score) of 1.93, below the threshold for species‐level identification, suggesting that strain P8‐9^T^ was not represented in the existing database. Subsequent 16S rRNA gene sequencing revealed its highest similarities to *P. castrilensis* (97.82%) and *P. butanolivorans* (97.43%), both below the 98.65% cutoff typically used for species discrimination [20, 30]. In the ML phylogenetic tree, strain P8‐9^T^ formed a distinct and strongly supported clade within *Peribacillus*, clearly separated from its nearest relatives (Figure 2).

**Figure 3:**
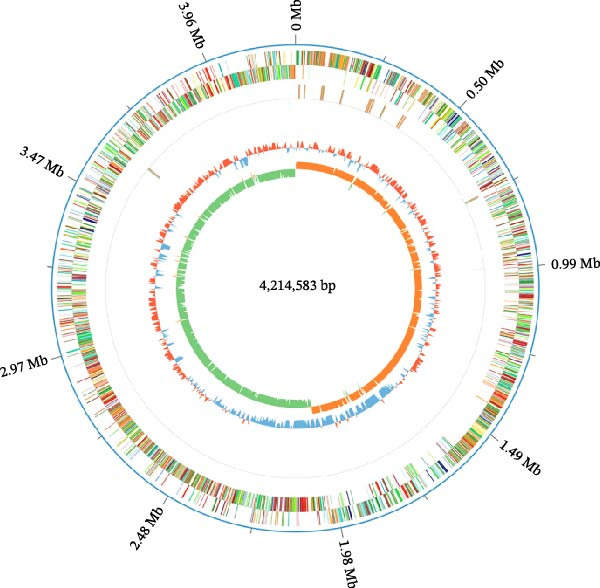
Complete genome architecture of *Peribacillus suis* sp. nov. Complete circular genome map of *P. suis* sp. nov. strain P8‐9^T^. From outer to inner rings: coding genes, noncoding RNAs (ncRNAs), GC‐content, and GC‐skew.

Whole‐genome sequencing produced a single circular chromosome of 4,214,583 bp with a G + C content of 40.95% (Figure 3). Comparative genomic analyses confirmed its novelty: ANI values between strain P8‐9^T^ and all type strains of recognized *Peribacillus* species ranged from 66.90% to 79.88%, while dDDH values ranged from 20.10% to 26.90% (Table 3). Both measures fall far below species thresholds (95%–96% ANI; 70% dDDH) [17, 18], providing robust genomic evidence that P8‐9^T^ represents a previously uncharacterized species. We, therefore, proposed the name *P. suis* sp. nov. with P8‐9^T^ (CCTCC AB 2025189) designated as the type strain.

**Table 3:** Average nucleotide identity and digital DNA–DNA hybridization values between strain P8‐9^T^ and reference strains of the genus *Peribacillus*.

Strain	ANI (%)	dDDH % [C.I]
*P. frigoritolerans* DSM 8001^T^	78.59	22.60 [20.4%–25.1%]
*P. aracenensis* BBB004^T^	78.41	21.90 [19.7%–24.4%]
*P. castrilensis* N3^T^	78.43	22.00 [19.7%–24.4%]
*P. muralis* DSM 16288^T^	77.44	21.30 [19.1%–23.7%]
*P. butanolivorans* DSM 18926^T^	79.88	23.50 [21.2%–26%]
*P. simplex* DSM 1321^T^	78.69	22.50 [20.2%–24.9%]
*P. acanthi* L28^T^	66.90	25.60 [23.2%–28%]
*P. alkalitolerans* KCTC 33631^T^	70.07	23.80 [21.5%–26.3%]
*P. asahii* MA001^T^	72.50	20.10 [17.9%–22.5%]
*P. cavernae* L5^T^	71.20	21.40 [22.1%–26.9%]
*P. deserti* DSM 105482^T^	69.48	24.40 [22.1%–26.9%]
*P. endoradicis* T3‐5‐0‐4^T^	66.32	21.90 [19.7%–24.4%]
*P. faecalis* AGMB 02131^T^	69.40	24.00 [21.7%–26.4%]
*P. glennii* V44‐8^T^	70.92	22.60 [20.3%–25.1%]
*P. kribbensis* DSM 17871^T^	69.10	20.30 [18.1%–22.7%]
*P. loiseleuriae* FJAT‐27997^T^	71.81	23.00 [20.7%–25.4%]
*P. psychrosaccharolyticus* ATCC 23296^T^	71.52	21.70 [19.5%–24.2%]
*P. saganii* V47‐23a^T^	70.39	22.90 [20.6%–25.4%]
*P. sedimenti* SCS‐155^T^	70.71	26.90 [24.5%–29.4%]
*P. tepidiphilus* SYSU G01002^T^	70.18	23.90 [21.6%–26.4%]

### 3.3. Genomic Insights Into Metabolic Potential and Pathogenicity Traits

Functional annotation of the 4267 predicted coding sequences showed a strong enrichment in metabolic pathways (KEGG; Figure 4A), consistent with the strain’s broad phenotypic catabolic capabilities. Importantly, genome screening uncovered a total of 219 putative virulence factors (Supporting Information 2: Dataset S1). Among these, 114 (52.1%) have homologs previously documented in the VFDB or other bacterial pathogens (Figure 4B), underscoring their potential functional relevance in pathogenesis. Additionally, five antibiotic resistance genes (Supporting Information 3: Table S1), predicted to confer resistance to glycopeptide, phosphonic acid, and tetracycline, were identified. These features reveal a substantial virulence and survival toolkit, atypical for the genus *Peribacillus*.

Figure 4Functional genomic annotation of *Peribacillus suis* sp. nov. Functional annotation of coding genes in *P. suis* sp. nov. strain P8‐9^T^. (A) KEGG pathway annotation. (B) Annotation by VFDB virulence factor annotation. (C) CO category annotation.

**Figure:**
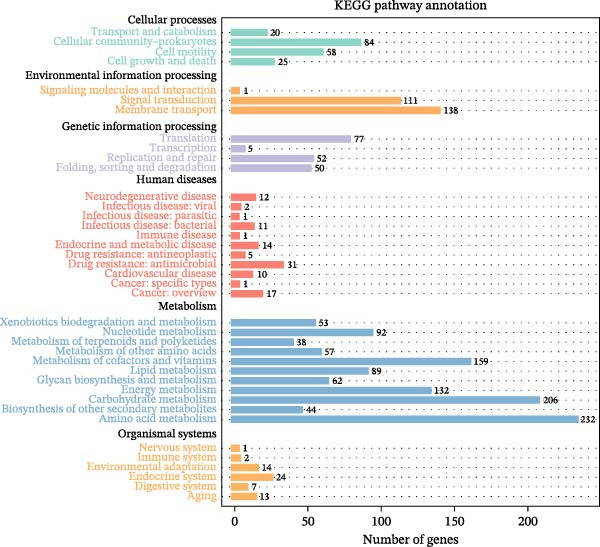
(A)

**Figure:**
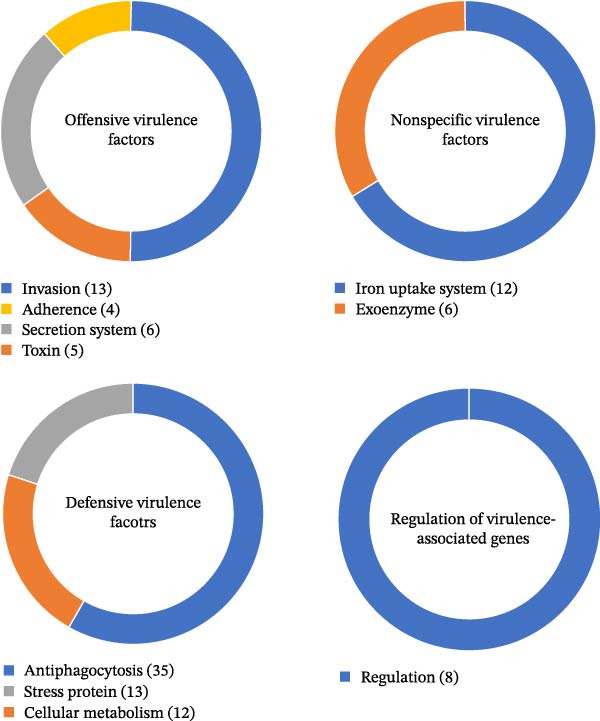
(B)

**Figure:**
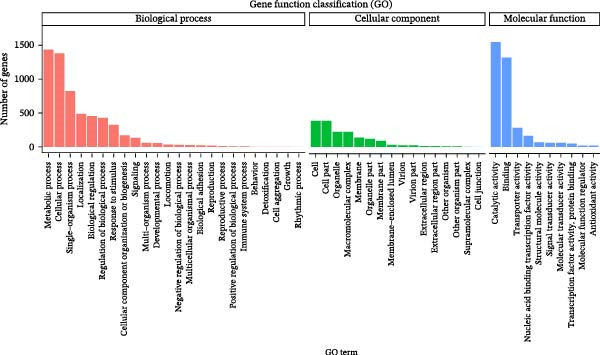
(C)

### 3.4. Pathogenicity Assessment in a Mouse Model

The pathogenic potential of *P. suis* sp. nov. was assessed in mice via two infection routes. IP inoculation induced rapid, severe systemic illness characterized by lethargy, anorexia, and all mice reached the humane endpoints within 24 h. Necropsy findings included pronounced intestinal congestion and abdominal effusion. Oral gavage (GW), in contrast, caused no mortality but produced marked splenomegaly (Figure 5A).

Figure 5Pathological and immunological responses to *Peribacillus suis* sp. nov. infection in mice. Impact of *P. suis* sp. nov. strain P8‐9^T^ infection on mouse pathology. Euthanasia was performed at 3, 9, and 24 h post‐infection or upon fulfillment of humane endpoint criteria, for sample and tissue collection (A) Gross morphology of major organs (liver, spleen, lungs, and duodenum). (B) Histopathology of infected organs. Cytokine concentrations in different tissues: (C) liver, (D) lungs, (E) spleen, and (F) duodenum. Magnifications: lung, liver, and spleen at 40X; duodenum at 20X (only collected from GW group). Statistical significance: ns; *p* > 0.05;  ^∗^ *p* < 0.05;  ^∗∗^ *p* < 0.01;  ^∗∗∗^ *p* < 0.001;  ^∗∗∗∗^ *p* < 0.0001.

**Figure:**
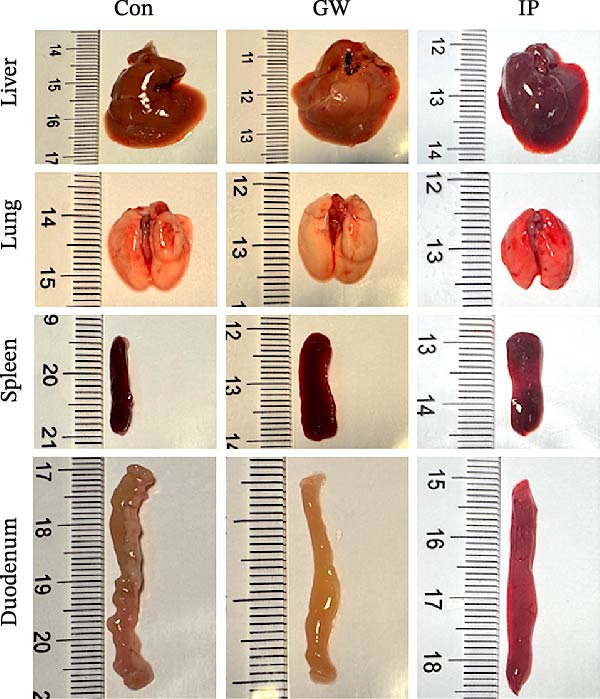
(A)

**Figure:**
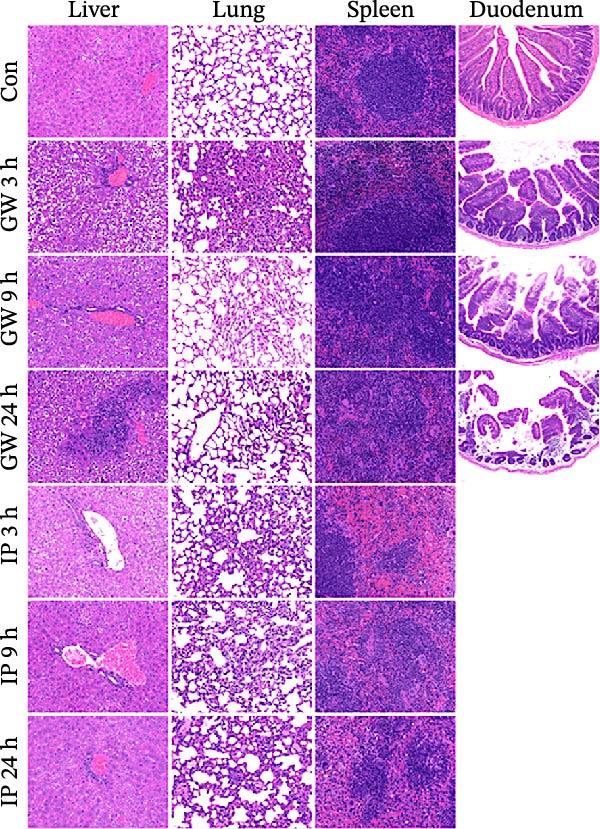
(B)

**Figure:**
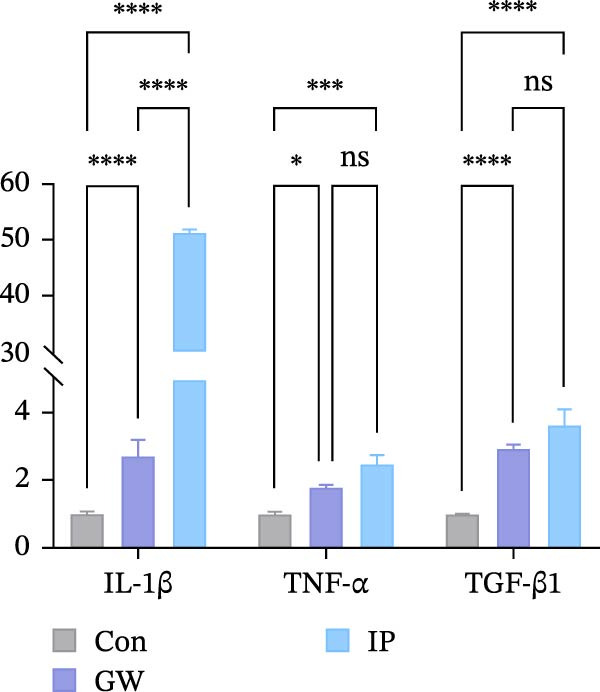
(C)

**Figure:**
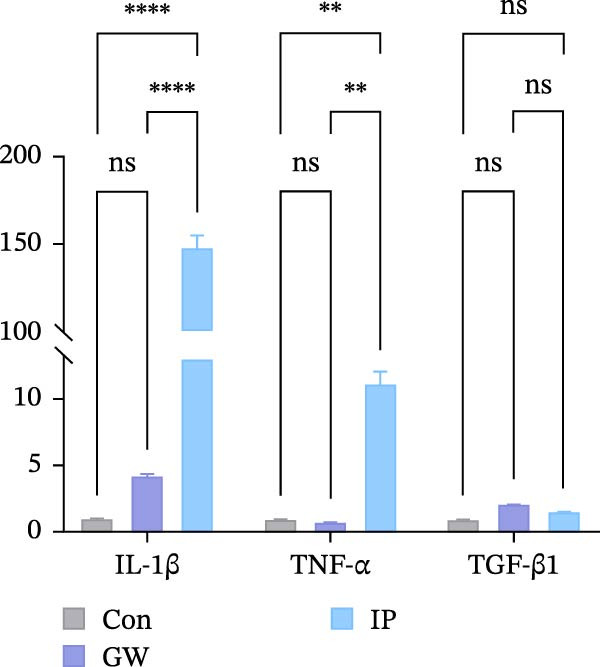
(D)

**Figure:**
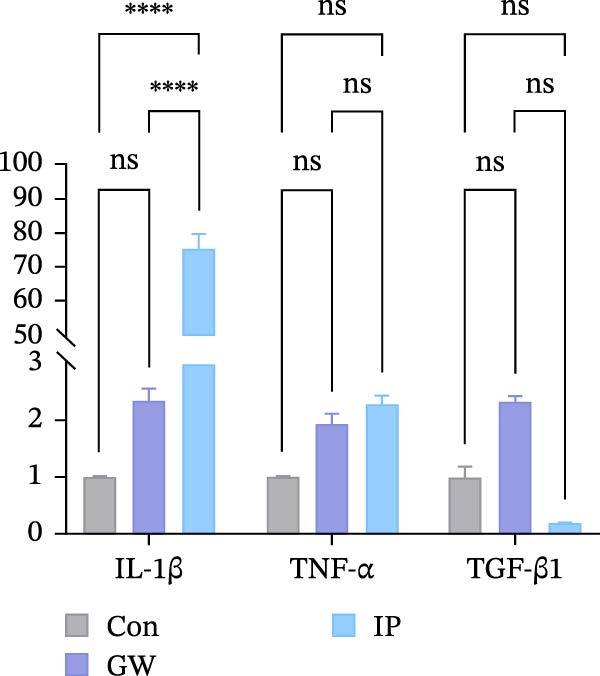
(E)

**Figure:**
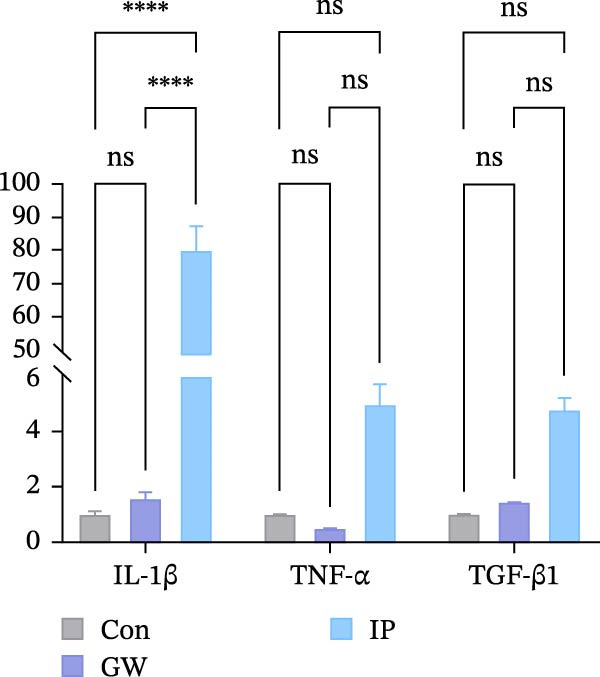
(F)

Histopathology revealed severe, time‐dependent multiorgan damage in IP‐infected mice (Figure 5B). The liver showed progressive hepatocellular necrosis with dense inflammatory infiltrates; the lungs exhibited collapsed alveolar structures and interstitial thickening; and the spleen displayed disrupted red–white pulp architecture with lymphocyte depletion. In GW mice, duodenal sections showed compromised villus morphology, culminating in extensive epithelial sloughing at 24 h.

These pathological changes were mirrored by a robust pro‐inflammatory transcriptional response. In the liver, IL‐1β and TNF‐α mRNA levels were significantly elevated in both infection groups compared with controls, with the IP group exhibiting the highest induction (Figure 5C–F). Significant upregulation of these cytokines was also detected in the lungs and spleen of IP‐challenged mice. In contrast, the GW group demonstrated a localized response, with no significant cytokine changes in the lungs or intestine.

## 4. Discussion

This study describes the discovery and characterization of *P. suis* sp. nov., a novel bacterial species isolated from the pig sucking louse *H. suis*. The primary motivation for specifically investigating the pathogenic potential of *P. suis* sp. nov. stems from a striking contrast between its ecological origin and the established profile of its genus. *Peribacillus* has been predominantly characterized as an environmental or plant‐associated genus with saprophytic or beneficial roles [8, 9]. The isolation of a putative member from the gut of a hematophagous ectoparasite, a niche far removed from soil or rhizospheres, presented an immediate and compelling scientific question: could this ecological shift be associated with a gain of function, specifically pathogenic capabilities? This focus was further justified by preliminary genomic screening, which revealed atypical abundance of putative virulence and antibiotic resistance genes in *P. suis* sp. nov., a feature not commonly reported in its benign relatives. Therefore, moving beyond mere taxonomic description to rigorously test its pathogenicity became a central objective of this study, aiming to probe whether the genus *Peribacillus* harbors underappreciated pathogenic lineages that emerge in specific host‐associated environments. Through a comprehensive polyphasic approach, we demonstrate not only its clear taxonomic novelty but also, the first direct experimental evidence of pathogenicity within the genus *Peribacillus*. These findings challenge the long‐standing view of this genus as uniformly benign.

The isolation of *P. suis* sp. nov. from an ectoparasitic arthropod markedly expands the known ecological scope of *Peribacillus*. Previously, members of this genus were almost exclusively associated with soil and plant environments, where they contribute to nutrient cycling and biological control [9–11]. Our findings reveal that *Peribacillus* can also persist in the highly specialized, hematophagous gut of an insect vector, a niche characterized by blood digestion, oxidative stress, and immune pressures. Such ecological shifts are often catalysts for the emergence of new pathogens, requiring substantial genomic and metabolic adaptation [31]. The unique environment of *H. suis* may, therefore, have driven the selection of the distinct virulence traits observed in *P. suis* sp. nov. This discovery underscores the potential of underexplored arthropod microbiomes to harbor previously unrecognized bacteria with novel biological roles [2, 4].

Equally striking is the clear demonstration of pathogenicity. Although sporadic reports have implicated *P. simplex* in opportunistic human infections [13, 14], evidence has been largely circumstantial. By comparison, our results show that pure cultures of *P. suis* sp. nov. are capable of causing fulminant systemic disease, meeting key criteria for establishing pathogenicity. The genome of *P. suis* encodes an extensive array of 219 putative virulence factors along with multiple antibiotic resistance genes; a combination typically associated with emerging opportunistic pathogens [32]. We propose that the transition from a benign environmental ancestor to this pathogenic lineage may have been facilitated by horizontal gene acquisition or selective pressures within the arthropod gut [33, 34].

Our in vivo findings highlight this virulence at both physiological and molecular levels. The IP challenge led to rapid disease progression consistent with septicemia, resulting in clinical signs that met predefined humane endpoints within 24 h. Oral challenge, by contrast, produced localized gut pathology and pronounced splenomegaly, indicating that *P. suis* sp. nov. may act as an opportunistic enteric pathogen capable of triggering chronic or subclinical inflammation in a natural host. These outcomes align with the known role of the gut as both a barrier and a gateway for systemic invasion by enteric bacteria [35]. The dramatic induction of IL‐1β and TNF‐α strongly suggests that cytokine‐driven hyperinflammation contributes to the acute tissue damage and lethality observed, which is consistent with the central role of these cytokines in septic shock and acute inflammatory syndromes [36].

The identification of a virulent bacterium within *H. suis* carries substantial veterinary and potential One Health implications. As an established vector of swine pathogens [5, 6], *H. suis* may function not only as a passive carrier but as a mobile reservoir, a “biological syringe,” facilitating transmission of *P. suis* within and between herds. However, we acknowledge that our study, which reports a single isolate, cannot determine the prevalence, colonization dynamics, or ecological role (e.g., transient vs. stable symbiont) of *P. suis* sp. nov. within louse populations. It remains possible that this strain represents a rare acquisition or an incidental finding. Therefore, the “reservoir” hypothesis, while plausible given the mechanical vector competence of *H. suis* and the virulence of *P. suis* sp. nov., requires rigorous ecological validation. The presence of antibiotic resistance genes further raises concern, as arthropod microbiomes are increasingly recognized as hotspots for horizontal gene exchange [37]. Such environments may promote the mobilization of resistance determinants from environmental or commensal pools into pathogens that directly impact livestock health.

Several limitations of this study pave the way for future research. First, and most critically, the mouse model used here cannot directly recapitulate infection in the natural porcine host. Mice and pigs differ significantly in immune responses, gut physiology, and microbiome composition, which likely lead to distinct disease patterns and host–pathogen interactions. Therefore, controlled infection studies in pigs are essential to define the true pathogenicity and clinical significance of *P. suis* sp. nov. Second, the predicted virulence factors require functional validation. Approaches, such as transposon mutagenesis or CRISPR‐based screens, are needed to confirm their roles and elucidate the underlying molecular mechanisms of pathogenicity [38]. Third, while the acute pathology and mortality strongly implicate *P. suis* sp. nov., we acknowledge that we did not perform bacterial re‐isolation and molecular identification from the diseased mice to formally fulfill this aspect of Koch’s postulates in the present study. Future investigations should include this critical step to conclusively confirm the strain’s in vivo replication and direct causal role in the observed lesions. Fourth, our study employed a single high inoculum dose to establish clear pathogenic potential. Future quantitative studies are needed to determine median infectious (ID_50_) or lethal (LD_50_) doses, which would provide a more precise measure of virulence. Finally, our sampling was geographically limited. Broader surveys are necessary to determine the true prevalence, genetic diversity, and epidemiological significance of *P. suis* sp. nov. within *H. suis* populations.

## 5. Conclusion

The discovery of *P. suis* sp. nov. demonstrates that pathogenic bacteria can emerge from unexpected ecological niches, particularly within understudied arthropod vectors. This work highlights the value of integrating culturomics and genomics to uncover hidden microbial threats and raises urgent questions regarding the distribution, evolution, and host interactions of this newly identified pathogen. Addressing these questions will be essential not only for swine health management but also for understanding the broader principles of pathogen emergence and microbial evolution.

## Author Contributions

Guo‐Hua Liu, Yi‐Tian Fu, and Yan‐Yan Peng conceived and designed the study. Guo‐Hua Liu, Yi‐Tian Fu, and Ying Xun were responsible for sample collection. Yan‐Yan Peng, Yi‐Liu Liu, and Ya Zhang carried out the experiments. Yan‐Yan Peng, Yi‐Tian Fu, Shi‐Feng Hu, and Yuan‐Ping Deng analyzed the data and drafted the manuscript. Hany M. Elsheikha and Guo‐Hua Liu contributed to revising the manuscript.

## Funding

The study was funded by the National Natural Science Foundation of China (Grant 32473057), the National Key Research and Development Program of China (Grant 2024YFD1800103), the Science and Technology Innovation Program of Hunan Province (Grant 2025RC1053), and the Hunan Natural Science Foundation Youth Fund Project (Grant 2024JJ6548).

## Conflicts of Interest

The authors declare no conflicts of interest.

## Supporting Information

Additional supporting information can be found online in the Supporting Information section.

## Supporting information


**Supporting Information 1** Figure S1. Growth characteristics of *Peribacillus suis* sp. nov. strain P8‐9^T^. (A) NaCl tolerance a. (B) Growth at different temperatures. (C) Alkali (pH) tolerance ability.

**Supporting Information 2** Dataset S1. Complete list of 219 putative virulence factors identified in the genome of *Peribacillus suis* sp. nov. strain P8‐9^T^.

**Supporting Information 3** Table S1. Detailed annotation of five antibiotic resistance genes identified in the genome of *Peribacillus suis* sp. nov. strain P8‐9^T^.

## Data Availability

The 16S rRNA gene and complete genome sequences were deposited in GenBank under Accession Numbers PX287133 and JBQXXB000000000, respectively. Strain P8‐9^T^ has been deposited in the culture collection and is available under Accession Number CCTCC AB 2025189.
