# Supplementary figures and images for: Peribacillus suis sp. nov. Isolated From the Pig Louse Haematopinus suis Reveals Unexpected Pathogenic Potential in a Traditionally Benign Genus
Source: Transbound Emerg Dis. 2026 Mar 27;2026:8640992. doi: 10.1155/tbed/8640992 (PMC13372016; doi:10.1155/tbed/8640992)

**A**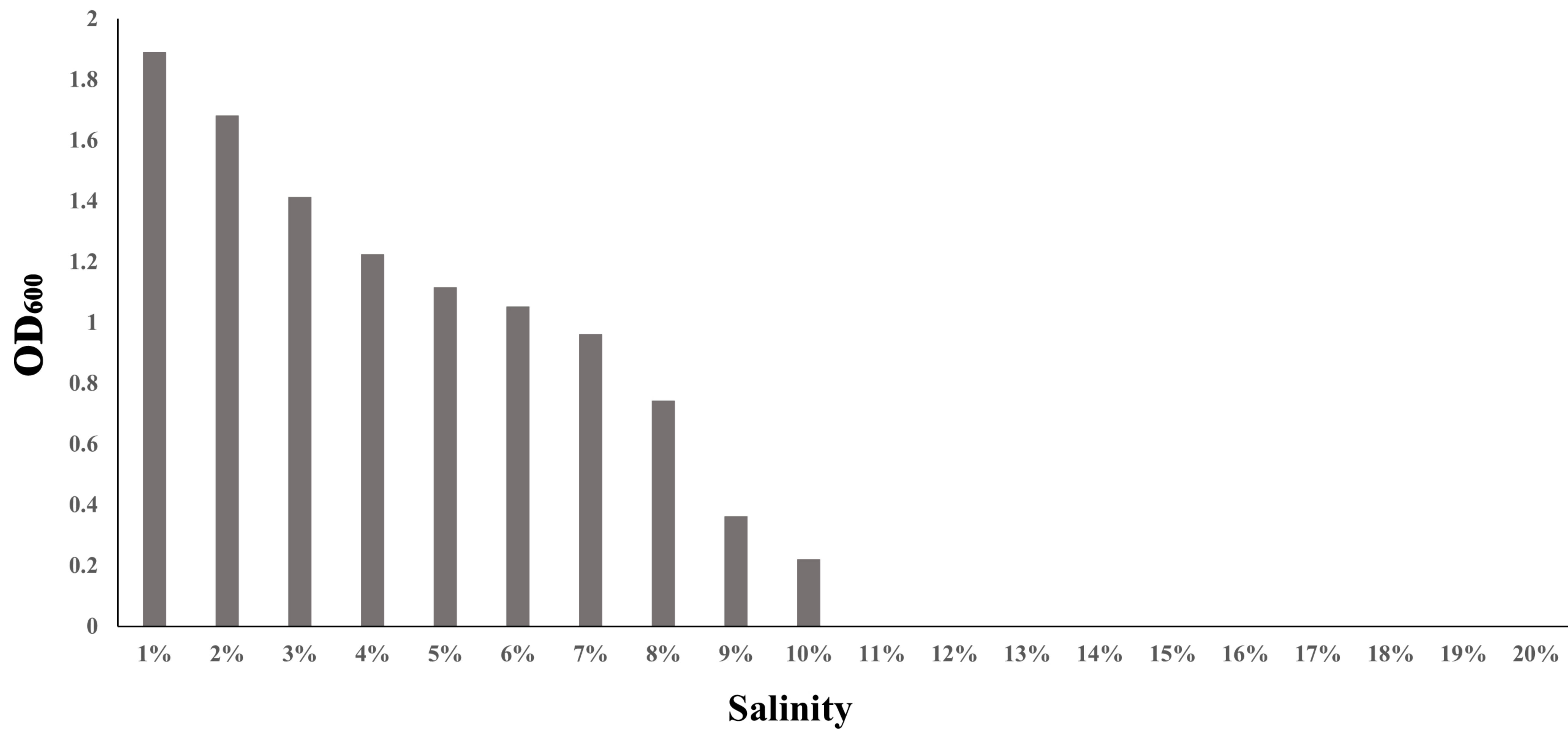**B**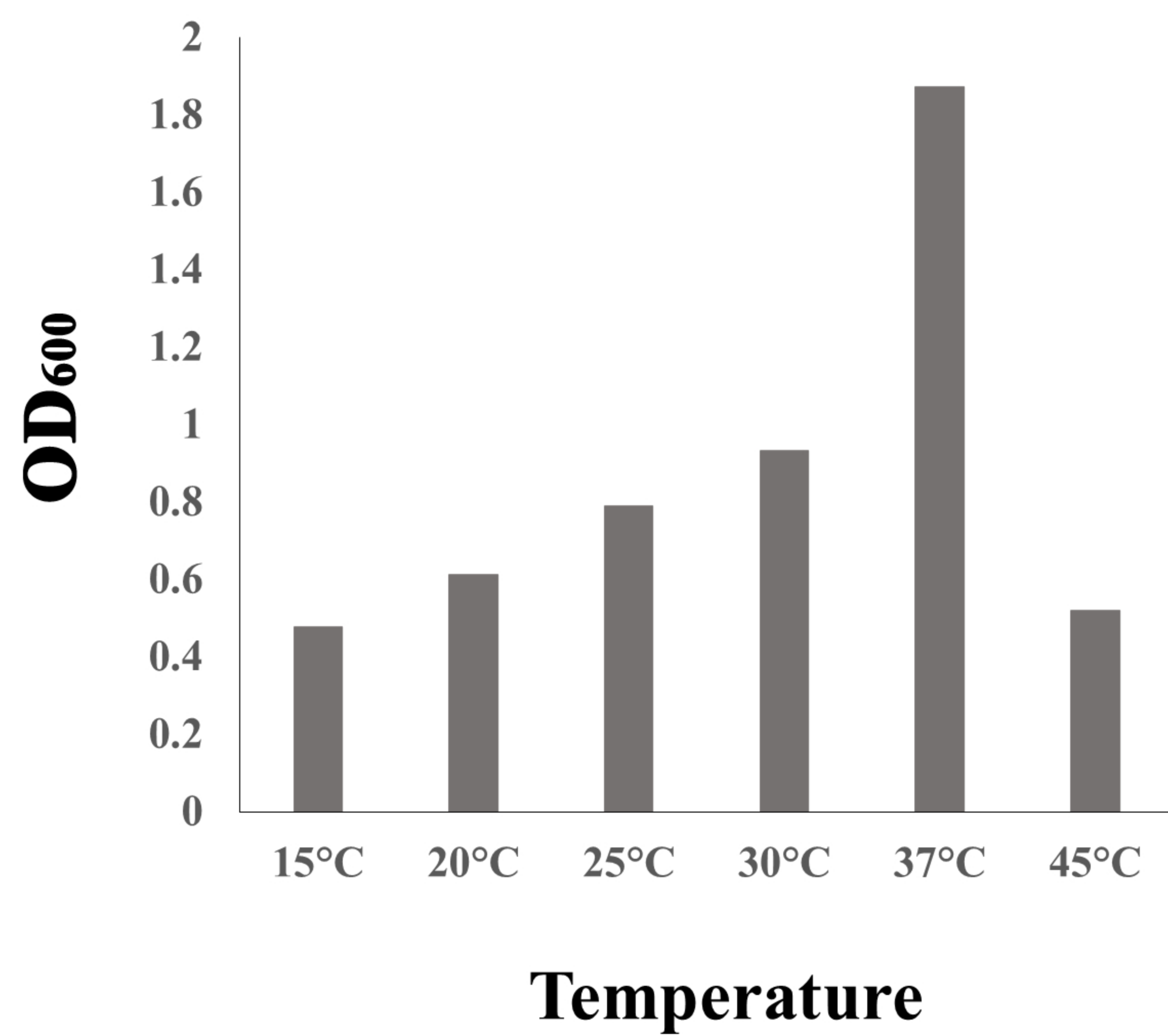**C**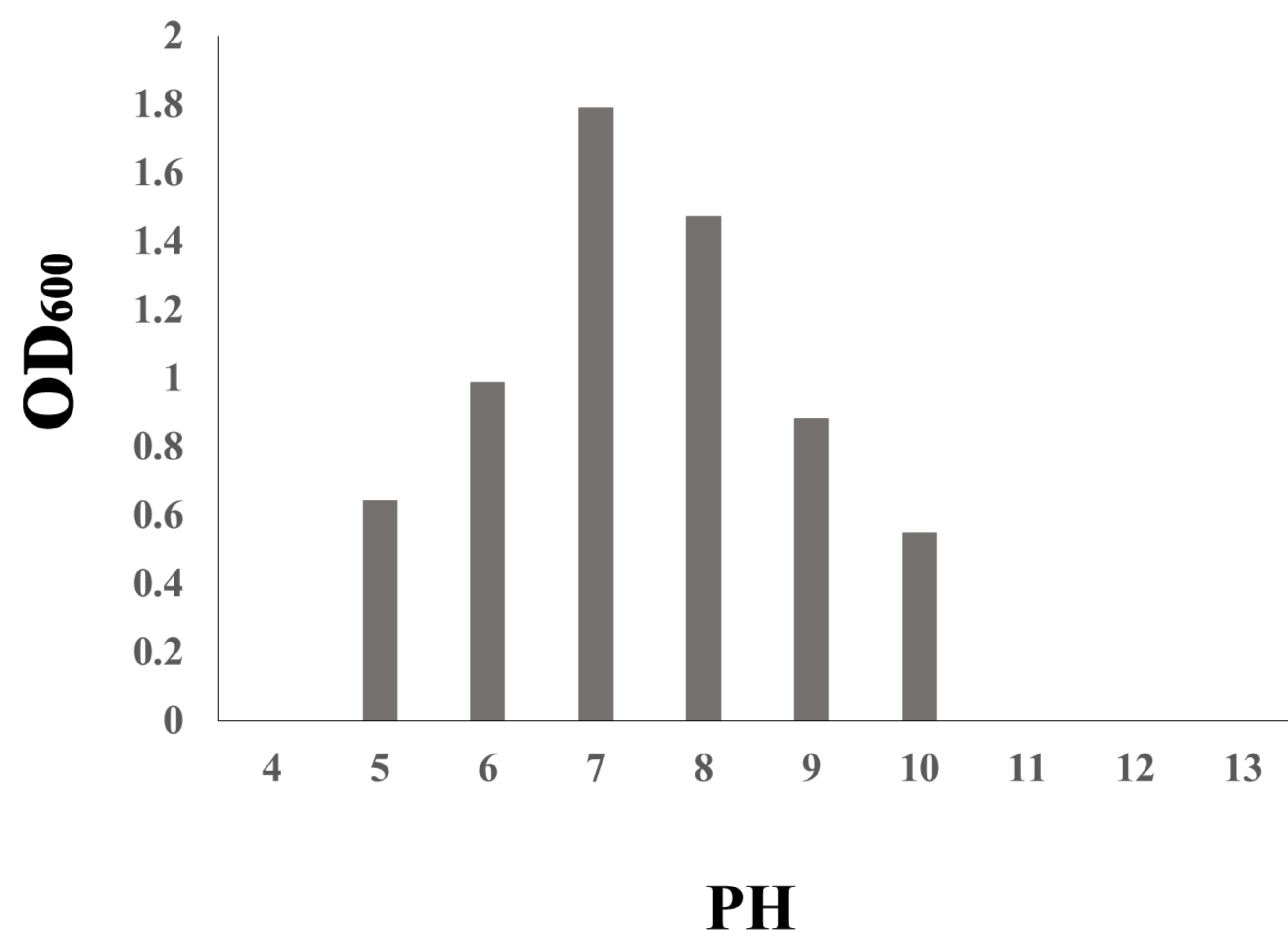

Supplement: Supplementary file 1 — Supporting Information 1 Figure S1. Growth characteristics of Peribacillus suis sp. nov. strain P8‐9T. (A) NaCl tolerance a. (B) Growth at different temperatures. (C) Alkali (pH) tolerance ability. [file TBED-2026-8640992-s001.pdf]
