# Supplementary material for: Peribacillus suis sp. nov. Isolated From the Pig Louse Haematopinus suis Reveals Unexpected Pathogenic Potential in a Traditionally Benign Genus
Source: Transbound Emerg Dis. 2026 Mar 27;2026:8640992. doi: 10.1155/tbed/8640992 (PMC13372016; doi:10.1155/tbed/8640992)
Supplement: Supplementary file 3 — Supporting Information 3 Table S1. Detailed annotation of five antibiotic resistance genes identified in the genome of Peribacillus suis sp. nov. strain P8‐9T. [file TBED-2026-8640992-s003.doc]

T_ABLE_ S1: Detailed annotation of five antibiotic resistance genes identified in the genome of *Peribacillus suis* sp. nov. strain P8-9^T^.

| **Gene_id** | **ARO_name** | **ARO** | **Drug_Class** | **Resistance_Mechanism** | **AMR_Gene_Family** |
| --- | --- | --- | --- | --- | --- |
| P_GM000284 | vanT gene in vanG cluster | 3002972 | glycopeptide antibiotic | antibiotic target alteration | glycopeptide resistance gene cluster; vanT |
| P_GM000475 | FosBx1 | 3007372 | phosphonic acid antibiotic | antibiotic inactivation | fosfomycin thiol transferase |
| P_GM000580 | vanW gene in vanI cluster | 3003724 | glycopeptide antibiotic | antibiotic target alteration | vanW; glycopeptide resistance gene cluster |
| P_GM002006 | tet(36) | 3000197 | tetracycline antibiotic | antibiotic target protection | tetracycline-resistant ribosomal protection protein |
| P_GM003912 | vanY gene in vanB cluster | 3002956 | glycopeptide antibiotic | antibiotic target alteration | vanY; glycopeptide resistance gene cluster |
